# Analysis of primary visual cortex in dementia with Lewy bodies indicates GABAergic involvement associated with recurrent complex visual hallucinations

**DOI:** 10.1186/s40478-016-0334-3

**Published:** 2016-06-30

**Authors:** Ahmad A. Khundakar, Peter S. Hanson, Daniel Erskine, Nichola Z. Lax, Joseph Roscamp, Evangelia Karyka, Eliona Tsefou, Preeti Singh, Simon J. Cockell, Andrew Gribben, Lynne Ramsay, Peter G. Blain, Urs P. Mosimann, Deborah J. Lett, Matthias Elstner, Douglass M. Turnbull, Charles C. Xiang, Michael J. Brownstein, John T. O’Brien, John-Paul Taylor, Johannes Attems, Alan J. Thomas, Ian G. McKeith, Christopher M. Morris

**Affiliations:** Edwardson Building, Institute of Neuroscience, Newcastle University, Campus for Ageing and Vitality, Westgate Road, Newcastle upon Tyne, NE4 5PL UK; Medical Toxicology Centre, Newcastle University, Wolfson Building, Claremont Place, Newcastle, NE2 4AA UK; Wellcome Trust Centre for Mitochondrial Research, Institute of Neuroscience, Newcastle University, The Medical School, Framlington Place, Newcastle upon Tyne, NE2 4HH UK; Bioinformatics Support Unit, Newcastle University, Leech Building, Framlington Place, Newcastle upon Tyne, NE2 4HH UK; University Hospital of Old Age Psychiatry, University Bern, CH 3010 Bern, Switzerland; Department of Neurology and Clinical Neurophysiology, Academic Hospital Bogenhausen, Technical University of Munich, Munich, Germany; Laboratory of Genetics at the National Institute of Mental Health/National Human Genome Research Institute, National Institutes of Health, Bethesda, Maryland MD20892 USA; Biomedical Research Building, Institute of Neuroscience, Newcastle University, Newcastle University, Westgate Road, Newcastle upon Tyne, NE4 5PL UK; Department of Psychiatry, University of Cambridge School of Clinical Medicine, Box 189, Level E4 Cambridge Biomedical Campus, Cambridge, CB2 0SP UK

**Keywords:** Dementia with Lewy bodies, α-synuclein, Primary visual cortex, Hallucinations, Alzheimer’s disease

## Abstract

Dementia with Lewy bodies (DLB) patients frequently experience well formed recurrent complex visual hallucinations (RCVH). This is associated with reduced blood flow or hypometabolism on imaging of the primary visual cortex. To understand these associations in DLB we used pathological and biochemical analysis of the primary visual cortex to identify changes that could underpin RCVH. Alpha-synuclein or neurofibrillary tangle pathology in primary visual cortex was essentially absent. Neurone density or volume within the primary visual cortex in DLB was also unchanged using unbiased stereology. Microarray analysis, however, demonstrated changes in neuropeptide gene expression and other markers, indicating altered GABAergic neuronal function. Calcium binding protein and GAD65/67 immunohistochemistry showed preserved interneurone populations indicating possible interneurone dysfunction. This was demonstrated by loss of post synaptic GABA receptor markers including gephyrin, GABARAP, and Kif5A, indicating reduced GABAergic synaptic activity. Glutamatergic neuronal signalling was also altered with vesicular glutamate transporter protein and PSD-95 expression being reduced. Changes to the primary visual cortex in DLB indicate that reduced GABAergic transmission may contribute to RCVH in DLB and treatment using targeted GABAergic modulation or similar approaches using glutamatergic modification may be beneficial.

**Electronic supplementary material:**

The online version of this article (doi:10.1186/s40478-016-0334-3) contains supplementary material, which is available to authorized users.

## Introduction

Dementia with Lewy bodies (DLB) accounts for up to 20 % of all dementia cases at autopsy [1]. Clinically DLB is associated with at least two of the following three core clinical features: recurrent complex visual hallucinations (RCVH), fluctuating cognition and parkinsonism [2] or one of these and one supportive feature (neuroleptic sensitivity, abnormal dopaminergic imaging of striatum, or rapid eye movement sleep behaviour disorder) [3]. It is the presence of RCVH, however, that has been suggested to be one of the most characteristic features of the disorder, and is associated with increased patient and caregiver distress and greater likelihood of nursing home admission and hospitalisation [2].

The RCVH in DLB are described as being well formed and involve animals, people, and objects [4] and show some similarities with the visual hallucinations seen in Charles-Bonnet syndrome and those following occipital infarction [5, 6]. Illusions and misperceptions are also experienced, and the occurrence of passage and presence hallucinations are also common, as with Parkinson’s disease (PD) and Parkinson’s disease with dementia (PDD) [7–9]. Whilst RCVH are one of the core clinical symptoms of DLB, their aetiological basis is essentially unknown. DLB subjects do show hypoperfusion and glucose hypometabolism in the medial occipital lobe including the primary and secondary visual cortices [10–12], which is associated with the visuoperceptual problems common to both DLB and PDD and may lead to misidentification or misinterpretation of objects and images [13]. Such visuoperceptual problems may indicate parietal and occipital dysfunction and relate to the RCVH experienced in DLB. RCVH have been suggested to be due to cortical atrophy, although most neuroradiological studies do not find significant atrophy of the medial occipital cortex in DLB [14–17] or in PD or PDD [14, 15, 18]. However, some studies show cortical thinning in the cuneus, precuneus and pericalcarine regions in DLB [19]. Whilst there appears to be involvement of the lateral occipital cortex in DLB [11, 12], the only significant pathology reported is spongiform change and gliosis in the medial occipital white matter [20], although this has not been observed in all studies [21, 22]. Ocular pathologies may also contribute to RCVH but these are common in elderly individuals, in addition to DLB and PD, where clouding, increased central and peripheral corneal opacity, and macular degeneration also occur [23, 24]. Changes in visual perception with decreased capacity to identify motion in spatial suppression tasks [25], and decreased motion perception [26, 27], along with reduced acuity [28] and ability to determine images in the peripheral fields are seen in older persons [29]. Changes in visual acuity are also common in DLB [13] and in PD [13, 23], although these are not more frequent than cognitively normal older individuals and the presence of retinal changes in PD, whilst suggested, has not been substantiated in some studies [30]. The interaction between reduced higher cortical activity and an altered proximal visual system associated with ageing may therefore contribute to the RCVH seen in DLB.

The well-formed nature of RCVH in DLB suggests that the ventral visual stream [31, 32] is affected. The ventral stream from the primary visual cortex projects to the temporal lobe, including the amygdala. These areas contain relatively high numbers of LB and it has been speculated that this may contribute to RCVH in DLB [33–35] although no differences between hallucinating and non-hallucinating cases have been observed. Nonetheless, high densities of Lewy bodies in the parahippocampal gyrus have been suggested to relate to early development of RCVH in both DLB [22] and also PD [24]. Alpha (α)-synuclein pathology in the retina in DLB [36] and cholinergic dysfunction in the medial occipital cortex in DLB [37] have also been observed indicating that pathological changes in the eye and biochemical changes in the primary visual cortex could potentially contribute to RCVH in DLB [38].

Since the possible changes in DLB that could contribute to RCVH do not stand alone but are part of the highly complex visual system, the question arises as to what are the molecular substrates of RCVH in DLB. Given the paucity of information on pathology in the primary visual cortex in DLB, but with the presence of a marked and specific hypometabolic deficit in this region in DLB [11, 39], it is possible that biochemical abnormalities in the visual cortex contribute to RCVH. We therefore explored this issue and describe here the analysis of the changes in the primary visual cortex in DLB using a combination of pathology and stereology to identify pathological and neuronal involvement, coupled with transcriptomic analysis of RNA and complimentary protein determination to determine the biochemical systems involved. A comparison group of individuals with Alzheimer’s disease (AD) was used to determine disease specific changes.

## Materials and methods

### Clinical samples

All procedures were approved by the Local Research Ethics Committee and tissue was obtained from the Newcastle Brain Tissue Resource (NBTR), a UK Human Tissue Authority approved Research Tissue Bank. The DLB and AD cases used in both pathological or biochemical series were from several local prospective clinical studies, with participants having clinical assessments repeated annually where appropriate until death and agreeing to donate tissue for research purposes. Clinical diagnoses of DLB and AD were made according to international consensus criteria [3] and NINCDS ADRDA criteria [40], respectively. Healthy control subjects were obtained from NBTR and all such cases had received thorough case note review to confirm they did not have cognitive impairment and had normal everyday function at time of death. The presence of eye disease was identified through case note review and in all cases this was identified by standard eye investigations. All cases had neuropathological confirmation of either having DLB, AD, or were neuropathologically normal according to established criteria [3, 41–44]. Cases of DLB had the presence of visual hallucinations documented by patient and informant interview through standard clinical interview according to consensus criteria [3] or were without any documented RCVH (1 case), whilst AD cases showed an absence of any prior history of RCVH. Tissue was obtained at post mortem, and the delay from death to freezing and fixation was noted. Tissue from the left hemisphere was rapidly frozen at -80 °C using pre-cooled copper blocks as approximately 1 cm thick slices and stored at -80 °C until use. The right hemisphere was fixed in 10 % formalin and processed by paraffin wax embedding.

For histopathological investigation, a 1 cm-thick coronal block of fixed tissue of the occipital lobe was cut into a series of 25 × 40-μm-thick tissue slices from a pre-defined paraffin-embedded coronal block containing the relevant portion of the middle of the primary visual cortex from a subset of patients (See Additional file 1: Table S1). Three of the 25 sections were sampled at equally spaced intervals (every eight sections) and stained with cresyl fast violet. The quality of each section was checked for consistency and the slides coded (CMM) to ensure that all analysis was carried out blind to diagnosis. The layers of the primary visual cortex were delineated and all measurements were taken from the cortical strip directly surrounding the calcarine sulcus. Additional paraffin sections (6–10 μm) were taken for pathological investigation and stained for myelin (Loyez stain), and with haematoxylin and eosin to show general structure. Immunohistochemical investigations of pathology used a standard ABC peroxidise method using anti-α-synuclein (KM1, Leica), anti-amyloid β peptide (4G8, Signet), and anti-phosphorylated tau (AT8, Thermo) and quantified using a standardised morphometric approach [45, 46] (antibody details given in Additional file 2: Table S6).

### Stereology

Neuronal density and volume was estimated within layers of the primary visual cortex using stereological analysis software (Stereologer, Chester, MD, USA) to apply the optical disector [47] and rotator [48] methods to obtain the density and volume estimates, respectively, as in our previous studies [49, 50]. Neurones were identified using standardised criteria: the presence of a Nissl-stained cytoplasm, pale nucleus and single identifiable nucleolus in cells that were not spherical unlike glial cells. The sections were viewed in oil using a Zeiss Photomicroscope at x 100 objective and a numerical aperture of 1.25. The microscope was attached to a JVC colour video camera TK-C1360B (JVC UK Ltd, London, UK), a motorised *x*- and *y*-axis stage accurate to 1 μm (Optiscan ES110, Prior Scientific Instruments Ltd, Cambridge, UK) and a Heidenhain *z*-axis depth gauge accurate to 0.5 μm (Heidenhain GB Ltd, London, UK) to ensure accurate measurement of disector depth.

Based on our previous investigations a random sampling strategy was used and estimates were conducted using one disector field per field of view and over 120 counts were made per layer examined in each subject to ensure precise estimates. Each disector frame measured 50 μm long, 50 μm wide and 15 μm deep, with a guard area of at least 4 μm taken (average μm) above and below the 3-D disector box, dependent on section thickness measurement. The mean coefficient of error (CE) for the overall neuronal and glial estimates were calculated using the Gundersen-Jensen method [51], as previously [49, 50] and showed that the mean CE for neuronal volume and density reached an acceptable level of precision in all layers in all cases measured (*P <* 0.15) [51]. There was no significant difference in demographic, clinical and histopathological information on the study sample (summarised in Additional file 1: Table S1). There were no significant differences between the groups in age, gender, tissue pH, duration of tissue fixation (d.f. = 25, *P* > 0.05 for all measures), or post-mortem interval. Mean (±SD) section thickness across all layers was comparable (d.f. = 25, *p =* 0.70) between control (31.82 [1.00] μm), AD (31.58 [0.79] μm) or DLB (31.89 [0.37] μm) groups after processing.

### RNA isolation

The primary visual cortex was identified in blocks of snap frozen tissue and approximately 50 mg of grey matter was dissected at -20 °C from a second series of 13 DLB, 12 control, and 13 AD cases with 12 DLB, 12 Control and 11 AD cases having RNA of sufficient quality for use and using identical clinical and pathological criteria to the group used for pathological investigation (see Additional file 3: Table S2) and placed in 5–10 volumes of pre-cooled RNA*later* solution (Ambion, Warrington, UK) and stored at -80 °C. Tissue was removed from RNA*later* and rapidly homogenised in TRI- Reagent (Ambion) and stored at -80 °C. RNA was extracted using a spin column method as per the manufacturer’s instructions (Ribopure, Ambion) and 1ug of RNA was DNase-treated (Turbo-DNAase free, Ambion). The RNA concentration was determined using a Nanodrop ND 1000 Spectrophotometer (Nanodrop Technologies) and RNA integrity number (RIN) examined with an Agilent 2100 Bioanalyzer RNA 6000 Nano Assay (Agilent Technologies, Stockport UK) according to the manufacturer’s instructions.

### Microarray analysis

Control (*n =* 12) and DLB (*n =* 12) total RNA samples were selected as a subset from the second group of patient material on the basis of tissue pH (>6.0) and RIN value (>6.0) (see Additional file 3: Table S2) and were analysed using Illumina Human-6 v2 BeadChips with approximately 48,000 transcript probes per chip using a GLP certified facility (Aros, Brensby, Denmark). Data was imported into R, log2 transformed using a Variance Stabilizing Transformation [52] and Robust Spline Normalisation algorithm designed for Beadarray data [53]. Following removal of failed samples and outliers using hierarchical clustering which excluded two array samples, significantly altered transcripts were identified in the limma package [54] and differentially expressed genes identified by applying cut-offs of *p*-Value <0.05 (Benjamini-Hochberg FDR correction [55]), and an absolute fold-change of greater than 2. To identify systems that may be altered/associated with DLB in the primary visual cortex we used Gene Ontology (GO) to identify biological process, molecular function and cellular component annotations using the GOstats package to test gene lists for over-represented terms [56] and also Kyoto Encyclopaedia of Genes and Genomes (KEGG) biochemical pathways.

### q-RT-PCR

Reverse transcription of RNA was performed on 1 μg of total RNA using oligo(dT)_12-18_ primer (Invitrogen, Warrington, UK), SupeRNase Inhibitor (Ambion), and Superscript III Reverse Transcriptase at 50 °C and samples stored at -80 °C until use. Semi-quantitative RT-PCR was performed in triplicate using Taqman® assays (see Additional file 4: Table S5; Applied Biosystems, Warrington, UK) using Taqman® Universal PCR Master Mix (Applied Biosystems). A total of 35 different brain tissue samples with total RNA of RIN > 6.0 were analysed by q-RT-PCR with 11 control, 12 DLB, and 12 AD subjects used. ABI PRISM® Sequence Detection System software was used to generate 2^-ΔΔCT^ values based on the comparative CT method with GAPDH mRNA as a reference [57]. To compare the relative gene expression, a Mann Whitney test was carried out using 2^-ΔΔCT^ values with significance level of *P <* 0.05.

### Protein determination

Tissue blocks from DLB (mean pH 6.3 ± 0.3), control (mean pH 6.3 ± 0.4), and AD cases (mean pH 6.0 ± 0.4) (see case details, Additional file 3: Table S2) of the occipital lobe were brought to -20 °C in a freezing cabinet and the primary visual cortex identified. Primary visual cortex tissue and a small amount of underlying white matter were isolated using a pre-cooled scalpel. Extraction was performed by homogenising 100–150 mg of tissue using a rotor stator type homogeniser in ice cold lysis buffer containing 0.2 M tetraethyl ammonium bicarbonate, pH 7.2 (TEAB; Sigma), 1 mM EDTA, and protease inhibitor tablets (Complete, Roche, Burgess Hill, UK) and samples stored at -80 °C. Samples were thawed, made to 0.02 % sodium dodecyl sulphate and sonicated on ice in a sonicating water bath for 20 min before protein quantitation was performed using a BCA Protein Assay kit (Pierce, Cramlington, UK).

Proteins (5–20 μg of protein per sample) were separated using NuPAGE 12 % Bis-Tris gels (Invitrogen) with 1x SDS NuPAGE MOPS Running Buffer containing NuPAGE Antioxidant (Invitrogen). Proteins in the gel were transferred to nitrocellulose membranes using an iBlot device (Invitrogen). Membranes were stained with Ponceau S solution (Sigma) to ensure equal protein loading, destained with 1x Tris-Buffered Saline (TBS) 0.2 % Tween 20, and stored in 1x TBS 0.2 % Tween 20 at 4 °C overnight. The membrane was incubated with 5 % dried milk in 1x TBS 0.2 % Tween 20 for 30 min at room temperature to block non-specific protein binding sites and incubated overnight with primary antibody (see Additional file 2: Table S6) diluted in 5 % dried milk in 1x TBS 0.2 % Tween 20 at 4 °C with agitation. Membranes were then washed for 10 min intervals 3 times with 1x TBS 0.2 % Tween 20 and incubated a further 30 min with the appropriate horseradish peroxidase conjugated secondary antibody at room temperature. Membranes were washed for 1 h with four washes of TBS 0.2 % Tween 20 then proteins visualised by Enhanced Chemiluminescence (GE, Amersham, Bucks, UK) and detected using x-ray film (Fuji, Fisher Scientific, Loughborough, UK). Protein bands were subsequently quantified using Image-J (NIH). Target protein expression was determined by normalising to GAPDH protein expression.

Validated commercial ELISA assays against BDNF (Promega Corporation, Madison, WI, USA), somatostatin, and neuropeptide Y (Cambridge Bioscience, Cambridge, UK) were used according to the manufacturer’s instructions. Samples of primary visual cortex tissue homogenates were thawed on ice and centrifuged at 16,000 x g for 20 min at 4 °C and the supernatant collected and diluted 1:20–1:50 in assay diluent buffer and applied to prepared assay plates. Samples were read along with standards using a Biotek Synergy Plate Reader. Protein in supernatant samples was determined using a BCA Protein Assay kit (Pierce) and specific peptide levels expressed as ng or pg peptide/mg protein.

### Statistical analysis

Analysis of pathology and stereology data involved analysis of variance (ANOVA), with pairwise comparison of differences and Bonferroni correction for multiple comparisons. PCR data was analysed using Mann Whitney U tests since the data were not normally distributed and protein data were analysed initially using Levine’s f-test to determine homogeneity of variance and differences using two-way unpaired t-tests. In all cases, *P <* 0.05 (uncorrected) was considered significant based on *a priori* data from microarray.

## Results

### Pathology

**Fig. 1:**
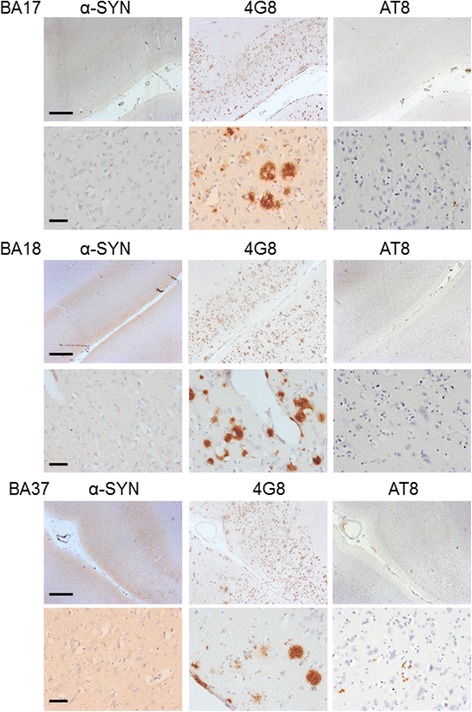
Neurodegenerative Disease Pathology in the Occipital Lobe in Dementia with Lewy Bodies. Representative staining for α-synuclein, Aβ (4G8), and hyperphosphorylated tau (AT8) in primary visual (BA17), secondary visual (BA18), and lateral occipital cortices (BA37) in DLB cases. An absence of α-synuclein pathology was seen in BA17, although increasing levels of α-synuclein could be seen in BA18 and in BA37. Similarly, an absence of AT8 (hyperphosphorylated tau) staining was seen in BA17 but increasing levels were seen in BA18 and BA37. Aβ (4G8) pathology was present in all cortical regions examined. Photomicrographs were taken at *x*2.5 magnification (upper rows) or at x40 magnification (lower rows) with scale bars at 1000 μm (upper rows) or 50 μm (lower rows)

Pathological investigation of the primary visual cortex in relation to RCVH, showed no evidence of α-synuclein deposition as Lewy bodies or Lewy neurites within primary visual cortex in any DLB case, with some evidence of α-synuclein deposition in BA18 as Lewy neurites in DLB cases, and increased deposition as Lewy neurites and occasional Lewy bodies in the lateral occipital cortex (see Fig. 1). The mean intensity of α-synuclein staining in three occipital regions was recorded for the primary visual, secondary visual and lateral occipital cortices. No difference was seen in staining intensity between DLB, AD and control cases for overall α-synuclein staining intensity. There was no statistically significant difference between the three areas in DLB compared to AD (ANOVA: F = (2,147) = 1.328, *p =* 0.268). A difference in the intensity of staining was seen between the primary visual, secondary visual and lateral occipital cortices within the DLB group (ANOVA: F[2,42] = 7.444, *p =* .002), with significantly more staining in secondary visual (114.49 ± 2.84, *p =* 0.038) and lateral occipital (119.10 ± 3.05, *p =* 0.001) cortices, compared to primary visual cortex (104.99 ± 1.86). A similar significant difference between the areas was also seen in the AD group (ANOVA: F = (2,147) = 32.275, *P <* 0.001), with this being significant between the secondary visual and lateral occipital cortices in AD (*p =* 0.440). Transcripts for α-synuclein mRNA were not significantly altered in DLB compared to controls, and similarly protein analysis of α-synuclein monomer (18 kDa) showed no significant change in expression in DLB, and comparable levels of expression were observed between control and AD subjects (see Additional file 5: Table S3), consistent with previous studies [58].

Assessment of amyloid-β and tau pathology demonstrated that the primary visual cortex in AD showed a high level of amyloid-β pathology in the form of senile plaque and diffuse amyloid-β deposition, which was significantly higher than in controls and also higher than in DLB (*P <* 0.005 vs control; *P <* 0.05 vs DLB, see Additional file 6: Figure S1). Amyloid-β pathology was found in the occipital cortex in DLB, but was not higher than in cognitively normal controls. No changes in APP mRNA expression were seen in the primary visual cortex using q-RT-PCR in either DLB or AD compared to controls (see Additional file 5: Table S3). Tau pathology, as assessed by AT8 immunostaining, was present throughout the occipital lobe in AD cases as neurofibrillary tangles and neuropil threads  and was significantly higher in the primary visual cortex but was minimal or absent from control and DLB cases (*P <* 0.05 vs control; *P <* 0.05 vs DLB, see Additional file 6: Figure S1). No changes in MAPT (tau) mRNA expression were seen in the primary visual cortex using q-RT-PCR in DLB, or AD cases compared to controls (see Additional file 5: Table S3).

### Stereology

**Fig. 2:**
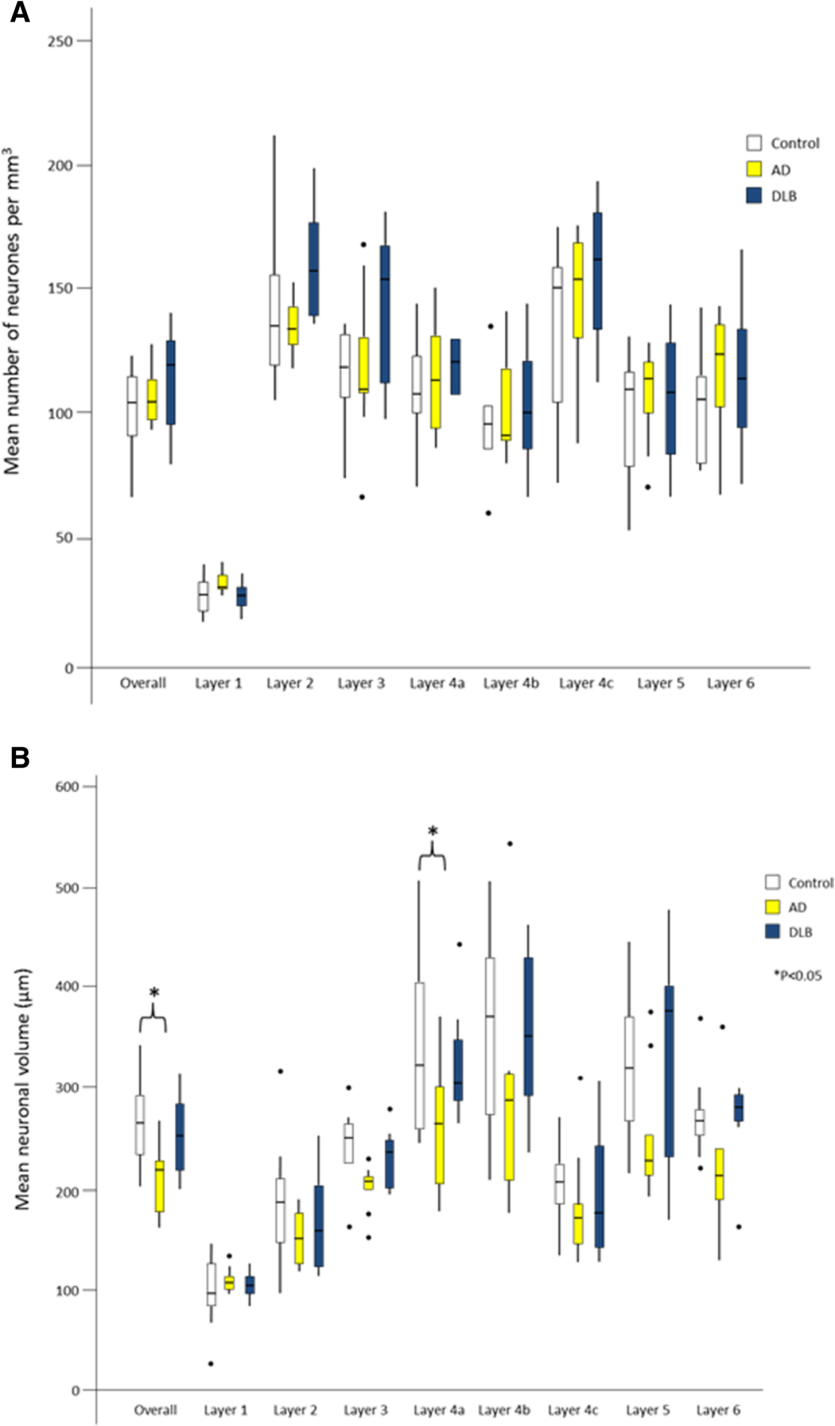
Neuronal density (**a**) and Neuronal volume (**b**) within the individual layers and across all layers (overall) of primary visual cortex in dementia with Lewy bodies  (DLB) and Alzheimer’s disease (AD). **a** Stereological analysis using the optical disector was used in primary visual cortex to determine neuronal density. No change in neuronal density was seen in DLB or AD. Boxplots show the median and quartile range with whiskers as the highest and lowest values within 1.5 x the interquartile range. ●, outlier points. **b** No overall reduction in neuronal volume was observed in DLB compared to elderly controls. There was a significant overall reduction in neuronal volume in AD compared to control and specifically a significant reduction in neuronal volume in layer 4a in AD (*P <* 0.05). Boxplots show the median and quartile range with whiskers as the highest and lowest values within 1.5 x the interquartile range. ●, outlier points. †, *P <* 0.05

Since neurodegenerative pathology in the primary visual cortex in DLB was not significantly different from that in normal elderly control individuals, neuronal densities were determined in the primary visual cortex. There were no significant differences in neuronal density in any layer of the primary visual cortex between control and DLB or AD groups after multiple comparison analysis (Fig. 2a). However, there was a significant reduction in neuronal volume in layer 4a in the AD group (*p =* 0.042; see Fig. 2b) in comparison to the control group. Furthermore, a significant reduction in neuronal volume in the AD (*p =* 0.043) versus control group was found across all layers when combined. No significant changes were found in neuronal volume in the DLB group against control or AD groups in any layer of the primary visual cortex. Similarly, since hypoperfusion and hypometabolism is a feature of DLB occipital cortex [11], we determined the presence of capillary density in primary visual cortex in DLB using Glut-1 staining. No alterations in capillary density were observed in either DLB or AD brain compared to age matched controls (not shown) suggesting hypoperfusion and hypometabolism is not associated with altered capillary density.

### Microarray

**Fig. 3:**
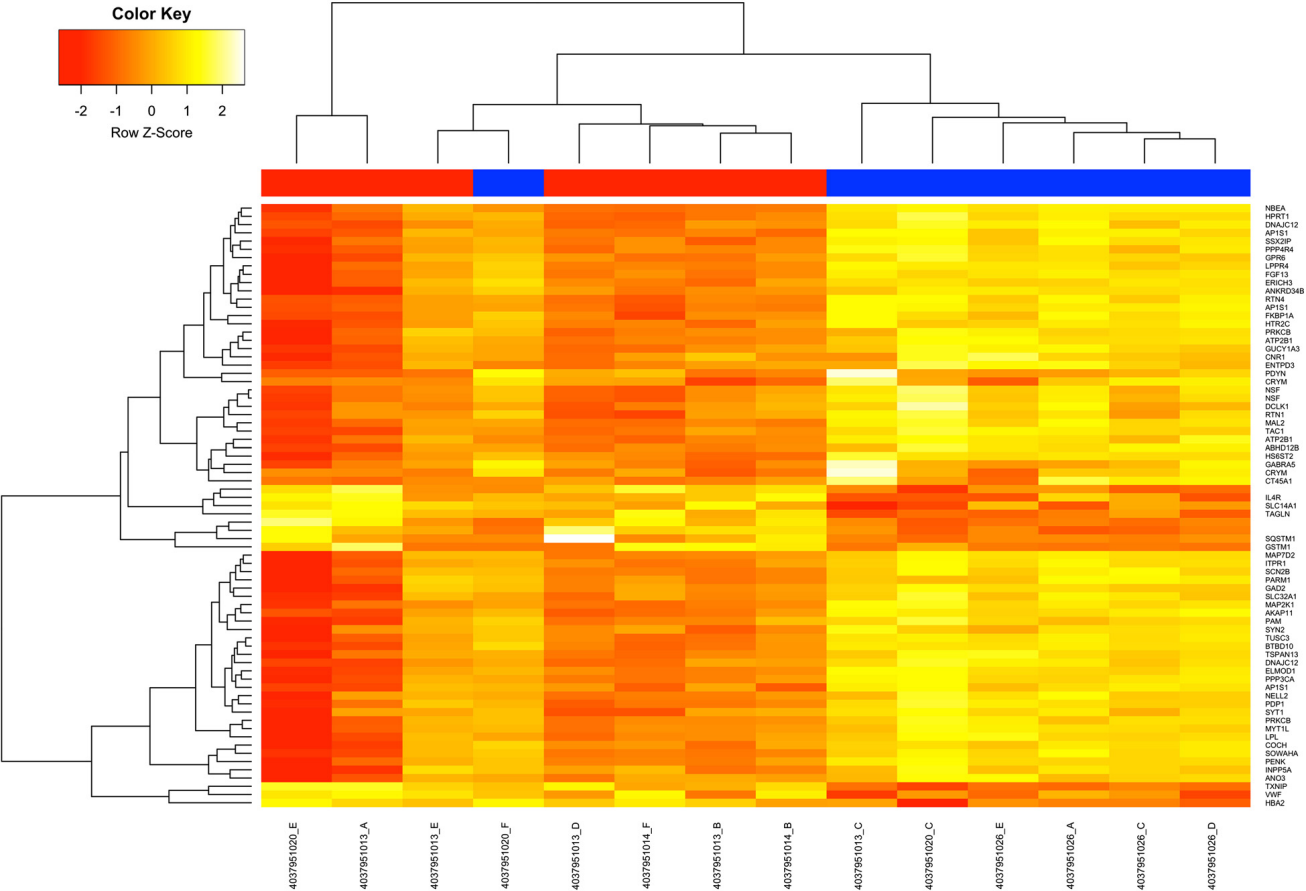
Microarray Analysis of Gene Expression in the Primary Visual Cortex in DLB. Total RNA samples were applied to Illumina whole genome transcript arrays and following analysis upregulated or downregulated transcripts analysed using hierarchical clustering and significantly altered genes were used to determine groups of genes associated with altered expression in DLB (Blue) compared to controls (Red) using a heatmap approach. DLB cases showed clustering with the exception of one DLB case (*) which had no known evidence of RCVH located within the control group

Owing to the lack of significant α-synuclein pathology or of major amyloid-β or Tau deposition in DLB and any significant cell loss in the primary visual cortex in DLB, whole genome microarrays were used to determine the changes in gene expression associated with hypoperfusion and hypometabolism. Seventy three genes were seen to be significantly altered in the primary visual cortex using the described cut-offs (see Fig. 3 and Table 1). Several systems were identified as altered in the primary visual cortex and linked with DLB most notably in cellular signalling, synaptic transmission, and vesicle transport (see Additional file 7: Table S7). However, GO-term analysis specifically identified GABAergic changes as being prominent (see Additional file 7: Table S7). For example, the mRNAs encoding neuropeptide precursor proteins such as proenkephalin (PENK), tachykinin (TAC1) and prodynorphin (PDYN) transcripts (see Table 1) were altered. These neuropeptides are primarily expressed by specific interneurone populations which are also GABAergic. This suggests there may be GABAergic dysfunction in the primary visual cortex in DLB, a hypothesis supported by the presence of other GABAergic markers in this patient group, most notably GAD2 (GAD65) and SLC32A1 and therefore we focussed on the GABAergic system. Using GAD65/67 to stain GABAergic cells, no alteration in total counts of cells was seen in the cortex of DLB or AD patients when compared to control in either primary visual (ANOVA: F[2,23] = 0.389, *p =* 0.682) or secondary visual cortices (ANOVA: F[2,23] = 1.184, *p =* 0.186; see Additional file 8: Table S4). This was paralleled by an absence of any change in *GAD1* mRNA transcripts in DLB relative to control, although a slight but significant reduction *GAD1* mRNA was seen in AD compared to control (*p =* 0.0337, uncorrected; Table 2), though AD and DLB groups were comparable. Similarly, there was no alteration in GAD65 or GAD67 protein determined by western blotting in either DLB or AD compared to control (see Table 2) suggesting that GABAergic neurones are still present in normal numbers but dysfunctional.

**Table 1:** Microarray analysis of Gene Expression in Primary Visual Cortex in DLB

symbol	name	logFC	adj.P.Val
**PENK**	**proenkephalin**	**2.328**	**0.003**
HTR2C	5-hydroxytryptamine (serotonin) receptor 2C, G protein-coupled	2.029	0.001
SOWAHA	sosondowah ankyrin repeat domain family member A	1.588	0.001
MAL2	mal, T-cell differentiation protein 2 (gene/pseudogene)	1.583	0.001
**TAC1**	**tachykinin, precursor**	**1.537**	**0.003**
FKBP1A	FK506 binding protein 1A, 12 kDa	1.527	0.001
**PRKCB**	**protein kinase C, beta**	**1.489**	**0.003**
ENTPD3	ectonucleoside triphosphate diphosphohydrolase 3	1.452	0.022
ELMOD1	ELMO/CED-12 domain containing 1	1.436	0.001
RTN4	reticulon 4	1.409	0.001
ATP2B1	ATPase, Ca++ transporting, plasma membrane 1	1.388	0.003
GPR6	G protein-coupled receptor 6	1.387	0.003
**PPP3CA**	**protein phosphatase 3, catalytic subunit, alpha isozyme**	**1.372**	**0.002**
COCH	cochlin	1.355	0.005
CRYM	crystallin, mu	1.334	0.035
**SLC32A1**	**solute carrier family 32 (GABA transporter), member 1**	**1.329**	**0.010**
PRKCB	protein kinase C, beta	1.318	0.006
AP1S1	adaptor-related protein complex 1, sigma 1 subunit	1.297	0.001
**GUCY1A3**	**guanylate cyclase 1, soluble, alpha 3**	**1.285**	**0.004**
FGF13	fibroblast growth factor 13	1.265	0.001
SYT1	synaptotagmin I	1.243	0.009
**GABRA5**	**gamma-aminobutyric acid (GABA) A receptor, alpha 5**	**1.236**	**0.020**
CT45A1	cancer/testis antigen family 45, member A1	1.230	0.047
LPL	lipoprotein lipase	1.226	0.007
MAP7D2	MAP7 domain containing 2	1.199	0.004
**PDYN**	**prodynorphin**	**1.196**	**0.046**
TSPAN13	tetraspanin 13	1.182	0.003
PDP1	pyruvate dehyrogenase phosphatase catalytic subunit 1	1.180	0.003
TUSC3	tumor suppressor candidate 3	1.164	0.001
NELL2	NEL-like 2 (chicken)	1.161	0.010
SSX2IP	synovial sarcoma, X breakpoint 2 interacting protein	1.153	0.001
SYN2	synapsin II	1.151	0.009
ITPR1	inositol 1,4,5-trisphosphate receptor, type 1	1.150	0.003
AP1S1	adaptor-related protein complex 1, sigma 1 subunit	1.147	0.001
AP1S1	adaptor-related protein complex 1, sigma 1 subunit	1.139	0.003
**GAD2**	**glutamate decarboxylase 2 (65 kDa)**	**1.132**	**0.014**
PPP4R4	protein phosphatase 4, regulatory subunit 4	1.125	0.002
ANKRD34B	ankyrin repeat domain 34B	1.123	0.003
ATP2B1	ATPase, Ca++ transporting, plasma membrane 1	1.117	0.005
NSF	N-ethylmaleimide-sensitive factor	1.112	0.002
BTBD10	BTB (POZ) domain containing 10	1.105	0.001
SCN2B	sodium channel, voltage-gated, type II, beta subunit	1.099	0.003
NSF	N-ethylmaleimide-sensitive factor	1.087	0.002
RTN1	reticulon 1	1.083	0.005
PARM1	prostate androgen-regulated mucin-like protein 1	1.078	0.010
ANO3	anoctamin 3	1.076	0.029
CRYM	crystallin, mu	1.072	0.030
**CNR1**	**cannabinoid receptor 1 (brain)**	**1.072**	**0.039**
LPPR4	lipid phosphate phosphatase-related protein type 4	1.069	0.001
MAP2K1	mitogen-activated protein kinase kinase 1	1.065	0.001
AKAP11	A kinase (PRKA) anchor protein 11	1.054	0.001
INPP5A	inositol polyphosphate-5-phosphatase, 40 kDa	1.043	0.027
**PAM**	**peptidylglycine alpha-amidating monooxygenase**	**1.041**	**0.006**
ERICH3	glutamate-rich 3	1.041	0.003
HPRT1	hypoxanthine phosphoribosyltransferase 1	1.030	0.001
ABHD12B	abhydrolase domain containing 12B	1.025	0.002
NBEA	neurobeachin	1.024	0.002
HS6ST2	heparan sulfate 6-O-sulfotransferase 2	1.021	0.001
DNAJC12	DnaJ (Hsp40) homolog, subfamily C, member 12	1.011	0.001
DCLK1	doublecortin-like kinase 1	1.010	0.018
DNAJC12	DnaJ (Hsp40) homolog, subfamily C, member 12	1.000	0.002
SLC14A1	solute carrier family 14 (urea transporter), member 1	−1.020	0.033
IL4R	interleukin 4 receptor	−1.071	0.039
GSTM1	glutathione S-transferase mu 1	−1.090	0.025
SQSTM1	sequestosome 1	−1.098	0.044
VWF	von Willebrand factor	−1.101	0.039
NPIPB4	nuclear pore complex interacting protein family, member B4	−1.141	0.004
TAGLN	transgelin	−1.178	0.004
LOC285407	asparagine-linked glycosylation 1 homolog pseudogene	−1.184	0.001
TMEM137	TMEM137	−1.244	0.002
HBA2	hemoglobin, alpha 2	−1.382	0.043
TXNIP	thioredoxin interacting protein	−1.522	0.003

Listing of significantly altered genes (±2.0 fold change, FDR < 0.05) from the whole genome microarray are displayed along with their average fold change. Neuropeptide/GABAergic interneurone markers are highlighted in bold

**Table 2:** GABAergic Interneurone Markers and Glutamatergic Markers in the Primary Visual Cortex in Dementia with Lewy Bodies and Alzheimer’s disease

Protein/mRNA	Control	DLB	AD
*GAD1* mRNA	1.46 ± 0.62	0.86 ± 0.22	0.62 ± 0.15*
GAD67 protein	0.85 ± 0.21	0.85 ± 0.16	0.72 ± 0.13
GAD65 protein	1.12 ± 0.26	1.16 ± 0.26	0.91 ± 0.15
*PVALB* mRNA	1.59 ± 0.47	0.87 ± 0.24†	3.51 ± 0.55**
PVALB protein	0.70 ± 0.22	0.61 ± 0.25*	0.87 ± 0.25***
NPY pg/mg	5.71 ± 1.95	6.63 ± 1.62	7.39 ± 1.52*
SST ng/mg	0.42 ± 0.18	0.48 ± 0.15	0.60 ± 0.27†
GABARAP-17 kDa	1.56 ± 0.28	0.91 ± 0.19***	1.29 ± 0.28**
GABARAP-14 kDa	1.47 ± 0.27	0.99 ± 0.14***	1.15 ± 0.26**
Gephyrin	1.13 ± 0.27	0.84 ± 0.08**	0.67 ± 0.09**
GABA A α1	0.75 ± 0.30	0.82 ± 0.26	0.83 ± 0.39
Kif5A	0.95 ± 0.58	0.51 ± 0.10†	0.90 ± 0.09
*GRIN2A mRNA*	1.44 ± 0.79	0.70 ± 0.16	0.89 ± 0.31
GluR1/GRIA1 protein	0.94 ± 0.13	0.89 ± 0.33	NT
vGlut1(SLC17A7) protein 61.3 kDa	1.20 ± 0.24	1.04 ± 0.18	0.91 ± 0.11†
vGlut1(SLC17A7) protein 59.3 kDa	0.90 ± 0.13	0.87 ± 0.1	0.90 ± 0.12
*PSD95 (DLG4) mRNA*	3.33 ± 1.14	1.80 ± 0.61†	0.83 ± 0.26*
PSD-95 protein	1.25 ± 0.22	0.62 ± 0.19***	1.24 ± 0.28
GAP43 protein	1.25 ± 0.45	0.71 ± 0.22***	1.08 ± 0.16
*BDNF mRNA*	0.70 ± 0.16	0.39 ± 0.14*	0.71 ± 0.48
BDNF protein	ND	ND	ND
*Synaptophysin (SYP) mRNA*	1.76 ± 0.72	0.89 ± 0.22	2.69 ± 0.80
Synaptophysin protein	0.96 ± 0.25	0.54 ± 0.09***	0.72 0.16*
*SNAP25 mRNA*	1.27 ± 0.30	0.88 ± 0.23	0.83 ± 0.25
SNAP25 protein	1.10 ± 0.28	0.99 ± 0.13	1.10 ± 0.17

Relative expression levels (+/- SEM) for GABAergic interneurone marker transcripts and proteins in control, DLB and AD primary visual cortex were determined using validated Taqman assays by real time PCR and proteins determined using western blotting of the appropriate protein band with relative expression levels being normalised to GAPDH mRNA or protein. Values represent the 2^-ΔΔCT^ value for mRNA and protein/GAPDH for the specific protein. NPY and SST levels were determined using specific sandwich ELISA. Uncorrected *p* values are presented with, *,* P <* 0.05 relative to control; **, *P <* 0.01 relative to control; ***,* P <* 0.005 relative to control; †, *P <* 0.1, >0.05. BDNF protein was not detected (ND) in any samples using an ELISA method

*NT* Not tested

### Protein

**Fig. 4:**
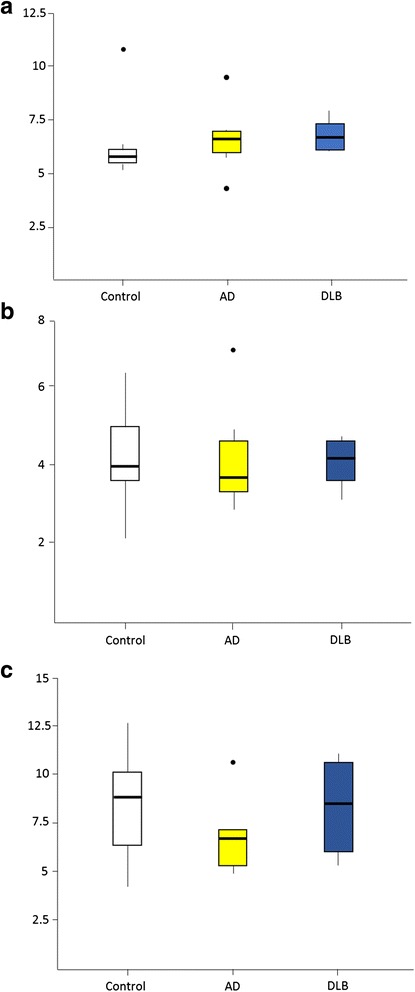
Analysis of Interneurone Populations in the Primary Visual Cortex in Dementia with Lewy Bodies and Alzheimer’s Disease. Immunohistochemistry for **a**) parvalbumin, **b**) calretinin, and **c**) calbindin was used to label specific interneurone populations in the primary visual cortex and neuronal density determined. No significant loss of interneurone populations was observed in either DLB or AD in the primary visual cortex. ^0^, outlier points

To explore the possibility that GABAergic neurones had either degenerated or were dysfunctional in specific neuronal subtypes, numbers of calcium binding protein containing neurones were quantified to determine if cell loss was selective. We studied the levels of parvalbumin (*PVALB*, mRNA; PVALB, protein), a marker of the fast spiking basket and chandelier neurones that provide major inhibitory inputs onto soma and axons of pyramidal neurones. *PVALB* mRNA was significantly reduced in DLB (*p =* 0.0035, uncorrected) compared to control but was also significantly increased in AD (*p =* 0.0002, uncorrected) (Table 2) compared to control. These changes in *PVALB* mRNA were reflected by changes in PVALB protein with a reduction of approximately 15 % in DLB (*p =* 0.033, uncorrected) and an increase in AD of approximately 25 % (*p =* 0.0005, uncorrected) when compared to control. Given the absence of any reduction in overall GABAergic neurones, parvalbumin, D-28 calbindin and calretinin neuronal density in the primary visual cortex was investigated using stereological methods. No significant change in the density of these neuronal markers in either DLB or AD were seen (see Fig. 4) suggesting that whilst the neurones may show altered marker profiles, their density is unchanged. To further investigate this, altered marker profile protein levels for other neuropeptides within the primary visual cortex in DLB were assessed. Neuropeptide Y levels, when compared to control, were not significantly increased on ELISA analysis in DLB by (*p =* 0.22, uncorrected) and in AD NPY was increased by approximately 20 % compared to control (*p =* 0.027, uncorrected) (see Table 2). Somatostatin levels in primary visual cortex were unchanged in DLB (*p =* 0.40, uncorrected) or in AD (*p =* 0.09, uncorrected) compared to control.

Since previous functional studies have suggested either decreased inhibition or increased excitatory drive in the primary visual cortex [59] and in light of the findings implicating GABAergic neurones based on the microarray data, we explored the possibility that altered inhibition was present. To determine if the neuropeptide changes were associated with alterations in other GABAergic markers, levels of several proteins involved in GABA neurotransmission were determined. The GABAergic post-synaptic marker GABARAP involved in GABA (A) receptor clustering/transport [60] showed reduced expression of the 17 kDa GABARAP isoform of approximately 40 % (*P <* 0.001, uncorrected) when compared to control and of the 14 kDa GABARAP isoform of approximately 30 % (*P <* 0.001, uncorrected) in DLB; in AD the 17 kDa isoform was reduced to a lesser extent by about 15 % (*P <* 0.001, uncorrected) and the 14 kDa isoform by 20 % (*P <* 0.001, uncorrected) when compared to controls (see Table 2). The GABA motor protein Kif5A showed a trend towards reduction in DLB compared to control (*p =* 0.06, uncorrected), but no change in AD. Gephyrin, a post synaptic GABA-A associated protein which anchors GABAergic and glycinergic receptors to the post-synaptic cytoskeleton [61] showed a significant reduction of approximately 25 % in DLB compared to control (*P <* 0.001, uncorrected) and in AD a reduction of about 20 % (*P <* 0.0001, uncorrected). Using ionotropic GABA A1 receptor subunit as an additional marker of receptor activity, showed no change in either DLB or AD when compared to control (see Table 2).

Since alterations of GABAergic activity may lead to altered net excitation, a series of glutamatergic synaptic markers were assessed (see Table 2). Using an anti-VGlut1 (SLC17A7) antibody as a marker of cortical input into the primary visual cortex, we demonstrated bands from 59–61 kDa, reflecting VGlut1 isoforms ranging from 59.3 to 61.3 kDa. DLB cases were not significantly different from controls in VGlut1 isoform 1 (61.3 kDa; *p =* 0.14), and AD cases were unchanged (*p =* 0.08, uncorrected). DLB cases were also not significantly different from controls in VGlut1 isoform 2 (59.3 kDa; *p =* 0.72) and AD cases were also not changed compared to controls (*p =* 0.78). Similarly the metabotropic glutamate receptor mGluR1 was also unaltered in DLB and we saw no loss of ionotropic AMPA glutamate receptor (GluR1/GRIA1) mRNA. As a marker of neural activity we observed a selective reduction in BDNF mRNA in the primary visual cortex in DLB cases when compared to control (*P <* 0.05, uncorrected; Table 2) but were unable to detect BDNF protein in any samples using a specific ELISA. Whilst variable, there was no change of the postsynaptic excitatory scaffold protein DLG4 (PSD-95) mRNA in DLB compared to control, and in AD this reduction was significant compared to control (*P <* 0.05, uncorrected; see Table 2). PSD95 protein determination, however, showed a marked 50 % decrease in DLB compared to controls (*p =* 0.0002, uncorrected), whilst AD cases showed no change in PSD95 (*p =* 0.96) in primary visual cortex compared to control. In line with PSD-95 findings, we identified a reduction of 43 % of the growth cone marker GAP43 (*p =* 0.0005, uncorrected), whilst AD cases showed no change (*p =* 0.11) when compared to control in DLB primary visual cortex. As markers of synaptic neurotransmission, we determined synaptophysin as a marker for presynaptic vesicular membranes and SNAP25 levels as a marker of presynaptic vesicles. Synaptophysin showed a marked and significant 44 % decrease compared to control in DLB cases (*P <* 0.00001, uncorrected) and also a lesser 22 % decrease in AD cases compared to control (*p =* 0.01, uncorrected). DLB cases, however, showed no change in SNAP25 expression compared to controls (*p =* 0.43), and similarly AD cases also showed no significant change in SNAP25 expression compared to controls (*p =* 0.13) indicating general exocytosis was unaltered.

## Discussion

Visual hallucinations have a complex aetiology with the potential involvement of multiple systems including the eye, primary visual cortex, and higher order brain regions [5, 6]. This study showed an absence of demonstrable neuropathological changes in primary visual cortex with no significant evidence of Lewy body- or AD- pathology or neuronal change in DLB, and similar to other studies [21], with the presence of mild pathology in adjacent occipital cortical regions. Recent evidence suggests that occipital involvement may be seen in individuals with PD with concomitant AD pathology, and also in cases with a clinical presentation of AD with the presence of Lewy bodies, although the presence of α-synuclein pathology within the primary visual cortex was not noted specifically [62]. This may indicate that the primary visual cortex could indeed be affected by pathology in DLB, if there are high levels of coexisting AD pathology, although in the current study such cases were not included. Whilst some studies show an absence of laterality in structural MRI in DLB [63, 64], some studies show lateralised changes [19], and therefore the current study, since it only determined cell counts and pathology in one hemisphere, may miss any unilateral changes. While the stereological approach used investigated all layers of the primay visual cortex, there may be a potential bias towards estimating pyramidal neurone changes since pyramidal neurones can be more readily identified in Nissl stained sections compared to small interneurones. The lack of any changes in any cortical layers enriched for pyramidal neurones in DLB would suggest no major alterations in pyramidal neurones due to oversampling. Additionally, the results from directly determining interneurone density changes which also appear unchanged in DLB (Fig. 4) suggest that any possible bias towards sampling large pyramidal neurones in the current study is likely to be small. The current results, however, suggest that occipital hypoperfusion and hypometabolism seen in conjunction with RCVH is not due to neurodegeneration within the primary visual cortex. Similar findings of reduced perfusion and metabolism including within occipital cortex in PD and in relation to visual hallucinations may indicate that the findings here in DLB may also relate to PD, although this will require further investigation [65–67]. In relation to the findings in AD, however, the presence of major pathology and also of neuronal atrophy, may indicate that the primary visual cortex is simply less responsive to stimulation, and consequently RCVH in AD are not common findings due to neurodegeneration.

Because of the absence of pathology in DLB, we used a transcriptomic approach to determine the gene expression changes that may underpin possible changes in the primary visual cortex. The current findings indicate there is an alteration in the profile of several genes and changes in GABA and neuropeptide markers indicate changes in the interneurone pool. Whilst the microarray findings show synaptic, and specifically GABAergic, changes, the major interneurone population is still intact based on the density of calcium binding protein positive neurones present, underscoring that dysfunction rather than degeneration occurs in the primary visual cortex in DLB. We have, however, not shown the presence of major changes in the levels of these calcium binding proteins and so it is difficult to determine if one specific group of interneurons is affected, although the microarray data appears to indicate that several groups of interneurons are affected, particularly enkephalinergic and tachykininergic neurones (see Table 1). Although the transcriptomic and morphological changes in the primary visual cortex point towards dysfunction, the underlying changes in neurotransmission that regulate this region require definition and synaptic changes associated with both inhibitory interneurones and excitatory neurones are present. Since RCVH are a very common feature of DLB (this study had access to only one case of DLB without RCVH; see Fig. 3) our findings may relate to an intrinsic property of the primary visual cortex in DLB, since while we used an AD group to look at degenerative changes, RCVH are not common in AD. The changes we have identified may therefore be a unique feature of DLB, rather than simply due to RCVH. The location of the individual case of DLB without RCVH in the control cluster following microarray would though indicate that the microarray findings may be linked to RCVH. Additional studies with cases of DLB where there is a well documented absence of hallucinations will be required to resolve this issue.

The current transcriptomic analysis points to altered synaptic function and specifically GABA dysfunction in DLB (see Additional file 7: Table S7). The changes in GABAergic interneurones suggest that there may be parallels with the changes seen in the primary visual cortex following reduced visual input in animals [68, 69]. Here, reduced input leads typically to functional changes in inhibitory interneurones with a reduced expression of parvalbumin in fast spiking interneurones [70]. There are also changes in the primary visual cortex following reduced stimulation with an increased turnover of synaptic boutons of interneurones both acutely and chronically [71, 72]. While much of this work has been conducted in the mouse, primate work also suggests a loss of GABAergic interneurone function following decreased input and this may occur acutely in man [73]. The hypometabolism seen in DLB in the medial occipital lobe in DLB may also relate to decreased GABA function since, studies support the possibility that blood flow is reduced with reduced GABA activity [74–76], although studies also indicate that increasing GABA levels causes reduced blood flow in younger subjects [77]. The reduced expression of GABAergic markers such as gephyrin and GABARAP may be an indication of the eye pathology frequently found in DLB patients and in ageing [13, 23]. DLB patients frequently show visuo-perceptual impairments and the presence of severe ocular pathology may reduce input to the primary visual cortex [13, 78] although the presence of eye disease is not a universal feature of DLB (Additional file 1: Table S1 and Additional file 3: Table S2). Conversely, environmental enrichment in mice leads to enhanced BDNF expression in the primary visual cortex [79], which contrasts with the potentially decreased BDNF mRNA expression seen in DLB cases in the current study (see Table 2). Visual input regulates the expression of BDNF mRNA [80] in glutamatergic neurones with reduction of BDNF leading to altered GABAergic transmission [81]. Changes within the eye [82], an absence of retinal thinning [83] and an absence of pathology within the lateral geniculate nucleus [84] indicate that not all DLB patients have eye disease (see Additional file 1: Table S1 and Additional file 3: Table S2) and this suggests that RCVH therefore do not require pathology within the eye or proximal visual system to occur or for hypometabolism and hypoperfusion to be present in the primary visual cortex. It is possible therefore that the findings indicate a stereotyped response of the primary visual cortex in an attempt to increase excitation by reducing post synaptic inhibitory synapses and decreasing inhibition, and would support the suggestion of decreased inhibition as a factor in RCVH in DLB [85]. Alternatively, the hypoperfusion and hypometabolism seen in the primary visual cortex in DLB may be due to lesions in other brain regions. Increased perfusion following cholinesterase inhibitor treatment occurs in lateral and posterior occipital cortex in DLB, and this may indicate that pathology within the basal forebrain cholinergic system may be in part responsible for the reduced perfusion observed [86]. Whilst there is a relatively selective reduction in perfusion of the primary visual cortex in DLB compared with AD, this is on a background of generalised hypometabolism involving temporal, parietal, and occipital regions when compared to elderly cognitively normal individuals [11, 87], and therefore other brain regions may potentially influence the perfusion and metabolism within the primary visual cortex.

This study indicates the presence of altered GABAergic synapses in DLB. Previous investigations of synaptic markers in the primary visual cortex in DLB have suggested loss of SNAP25 and syntaxin which we did not observe in the current study. In prior studies this may be due to the use of indirect ELISA determination with potential cross reactivity of antibodies, which would not be seen with our more robust western blotting approach [88]. Demographic factors, including longer post mortem interval and lower brain pH (indicating poor agonal state), are known to negatively affect synaptic protein levels [89] and will have influenced previous investigations. Our results are more in line with preserved function that would be seen with preserved SNAP25 levels, which is essential for neurotransmitter exocytosis [90]. Studies in other brain regions (e.g. frontal cortex) in DLB have shown relatively preserved synaptic markers [91, 92], which would correlate with the selective loss of certain marker proteins, rather than a generalised loss of synapses per se. The current results suggest connectivity is selectively altered in the primary visual cortex of DLB, with a change in specific synaptic marker proteins and genes, for example PSD95, compared to the atrophy associated changes seen with AD. PSD95 is specifically associated with the excitatory post-synaptic membrane [93]. Whilst the major input to the primary visual cortex from the lateral geniculate nucleus appears normal [84], input from adjacent visual cortical areas (secondary visual cortex, etc.) and through secondary visual pathways via the pulvinar occur, and so reductions in PSD95 may represent such defects. Using VGlut1 as a marker of cortico-cortical connections, we observed no significant reductions in VGlut1 in DLB but observed reductions in VGlut1 in AD where there was a general reduction along with atrophy in excitatory neurones (Fig. 2) due to pathology [94]. This would indicate that intrinsic and major cortical glutamatergic synapses are not lost in DLB but that changes within glutamatergic neurones may represent the observed changes in PSD-95. This supports the concept of reduced visual system input to the primary visual cortex as being part of the process of generation of RCVH in DLB as with other primary visual system lesions [5] and that “bottom up” changes are important in RCVH as has been noted for PD [95].

The changes in GABA neurotransmission may have a clinical correlate within the symptomatology of RCVH in DLB. Altered GABA neurotransmission may be observed as a change in the excitability of pyramidal neurones where in DLB, the use of transcranial magnetic stimulation of the occipital cortex (occiput) is correlated with increased generation of RCVH like phosphenes [96] possibly as a result of decreased inhibition. Reduced levels of parvalbumin, such as seen in this study, have been suggested to cause altered cortical gamma oscillations whereby the gain and modulation of firing of pyramidal neurones is affected [97, 98]. Desynchronisation of pyramidal neurones in the primary visual cortex and net increased excitability due to reduced inhibition may therefore lead abnormal outflow to higher order visual areas which appear to show changes in DLB [78] resulting in RCVH.

The presence of reduced visual input into the primary visual cortex in DLB, whilst being part of the aetiology of RCVH, does suggest that further changes must contribute to RCVH. For example, RCVH do not necessarily occur in blind individuals, and this suggests that other pathological or biochemical changes are required in order to result in RCVH [78]. Considerable attention has focussed on changes in temporal lobe structures in DLB and also PD in relation to visual hallucinations [7, 78]. Pathological correlates have indicated that temporal lobe Lewy bodies correlate with the presence of RCVH [33] and that, in PD, elevated Lewy body density in the basolateral nucleus of the amygdala and temporal lobe associate with RCVH [24, 34]. Structurally, however, there is relative preservation of the temporal lobe in DLB [17], although this may not be the case in PD with RCVH [99]. This is accompanied in DLB with decreased connectivity of the temporal lobe [78, 100] and perfusion deficits, specifically in the ventral occipitotemporal junction following visual stimulation of DLB patients with frequent and more severe RCVH [63].

Our results therefore show that, in the absence of major pathological changes in the primary visual cortex in DLB, there are functional changes associated with GABAergic neurones. This may reflect a change to the visual input due to reduced visual acuity as a consequence of age related changes in the eye. These changes lead to an inhibitory/excitatory imbalance in the primary visual cortex and altered perception of visual stimuli due to changes in association cortex. One possibility is that by treating major eye disease where it is present, by opting for a more proactive approach, RCVH may be reduced [101]. Alternatively, modulation of GABAergic inhibition using selective modulators of specific GABA receptors might be feasible to determine if RCVH are reduced in small scale clinical trials, using appropriate scales for estimating RCVH frequency and severity [63, 78]. Modulation of glutamatergic signalling may also be of benefit, perhaps by acting on group II metabotropic glutamatergic receptors, again in small scale experimental studies using appropriate scoring systems for RCVH [102]. It may therefore be possible to ameliorate the recurrent visual hallucinations perceived by DLB patients using appropriate interventions.

## Additional files

Additional file 1: Table S1. Patient Demographics of Neuropathology and Stereology Subjects [2, 3, 41]. (DOC 41 kb) Additional file 2: Table S6. Antibodies, suppliers, and usage. (DOC 47 kb) Additional file 3: Table S2. Case Details For Microarray, q-PCR and Biochemistry Series [2, 3, 41]. (DOC 41 kb) Additional file 4: Table S5. Taqman q-RT-PCR Assay Details used in determination of mRNA relative levels in primary visual cortex. Assay ID, specific assay identifier; Gene Name, mRNA target of assay. (DOC 41 kb) Additional file 5: Table S3. Analysis of pathology related genes in the primary visual cortex in Dementia with Lewy Bodies and Alzheimer’s disease. (DOC 30 kb) Additional file 6: Figure S1. Neurodegenerative Disease Pathology in the Occipital Lobe in (A) Control and (B) Alzheimer’s disease cases. Representative staining for α-synuclein, Aβ (4G8), and hyperphosphorylated tau (AT8) in primary visual cortex (BA17), secondary visual cortex (BA18), and lateral occipital cortex (BA37) in A) elderly normal control or in B) AD individuals. An absence of α-synuclein pathology was seen in BA17 in either AD or controls cases. Similarly, an absence of AT8 (hyperphosphorylated tau) staining was seen in BA17 in controls, but increasing levels were seen in BA17, BA18 and BA37 in AD. Aβ (4G8 antibody) pathology was present in all cortical regions examined with high levels seen in BA17 in AD cases and in other cortical regions examined. Photomicrographs were taken at *x*2.5 magnification (upper rows) or at x40 magnification (lower rows) with scale bars at 1000 μm (upper rows) or 50 μm (lower rows). (ZIP 1315 kb) Additional file 7: Table S7. Functional Systems altered in Primary Visual Cortex in Dementia with Lewy Bodies (DOC 42 kb) Additional file 8: Table S4. GAD65/67 Mean (± SD) Cell Counts of GABAergic Neurones in the Primary (BA17) and Secondary (BA18) Visual cortex in Dementia with Lewy Bodies and Alzheimer’s disease. (DOC 30 kb)
