# Supplementary material for: Analysis of primary visual cortex in dementia with Lewy bodies indicates GABAergic involvement associated with recurrent complex visual hallucinations
Source: Acta Neuropathol Commun. 2016 Jun 30;4:66. doi: 10.1186/s40478-016-0334-3 (PMC4928325; doi:10.1186/s40478-016-0334-3)
Supplement: Additional file 1: Table S1. — Patient Demographics of Neuropathology and Stereology Subjects [2, 3, 41]. (DOC 41 kb) [file 40478_2016_334_MOESM1_ESM.doc]

**Additional file 1: Table S1 Patient Demographics of Neuropathology and Stereology Subjects**

Age, age at death in years; PMI, post mortem interval (time from death to tissue fixation or freezing); pH, brain pH determined at post mortem on fresh brain tissue; Braak neurofibrillary staging according to (41). Compound Lewy body score is as described using consensus guidelines (2, 3).

|  | Control  N=10 | DLB  N=11 | AD  N=11 |
| --- | --- | --- | --- |
| Age (±SD)/ years | 77.60 (±8.0) | 76.46 (±6.6) | 77.64 (±4.9) |
| PMI (±SD)/ hours | 15.9 (±2.6) | 17.82 (±8.3) | 22 (±8.6) |
| Male/Female | 7/3 | 8/3 | 7/4 |
| pH (±SD) | 6.20 (±0.4) | 6.39 (±0.3) | 6.14 (±0.4) |
| Braak Stage(±SD) | 2.14 (±1.4) | 2.14 (±1.1) | 5.5 (±0.5) |
| Lewy Body Score (±SD) | 0 (n/a) | 7.91 (±8.1) | 0 (n/a) |
| Eye Disease* (positive/negative) | 1/9 | 6/5 | 3/8 |

†, Braak neurofibrillary tangle staging (41), data are presented as median and (range)

‡, Cortical Lewy body score (3), data are presented as median and (range)

*, significant eye disease: glaucoma, macular degeneration, bilateral cataracts, orbital exentoration.
