# Supplementary material for: Analysis of primary visual cortex in dementia with Lewy bodies indicates GABAergic involvement associated with recurrent complex visual hallucinations
Source: Acta Neuropathol Commun. 2016 Jun 30;4:66. doi: 10.1186/s40478-016-0334-3 (PMC4928325; doi:10.1186/s40478-016-0334-3)
Supplement: Additional file 2: Table S6. — Antibodies, suppliers, and usage. (DOC 47 kb) [file 40478_2016_334_MOESM2_ESM.doc]

**Additional file 2: Table S6** Antibodies, suppliers, and usage.

| **Product Name** | **Company** | **Code** | **Primary Dilution** | **Use** |
| --- | --- | --- | --- | --- |
| Kinesin 5A (Kif5A) | AbCam | ab154414 | 1:500 | WB |
| Gephyrin | Synaptic Systems | 147111 | 1:3000 | WB |
| PSD95 (7E3-1B8) | Thermo | MA1-046 | 1:50000 | WB |
| GABA-A-R-Alpha 1 | NeuroMab | N95/35-73-136 | 1:50 | WB |
| GABA A Receptor alpha 2 [N1C2] | GeneTex | GTX105282 | 1:1000 | WB |
| GAPDH (FL-335) | Santa Cruz | sc-25778 | 1:2000 | WB |
| Synaptophysin (SY38) | Sigma | N/A | 1:50000 | WB |
| SNAP-25 C terminal | Enzo | BML-SL3730 | 1:5000 | WB |
| GABARAP [EPR4805] | AbCam | ab109364 | 1:5000 | WB |
| GAP43 | Santa Cruz | sc-17790 | 1:500 | WB |
| alpha synuclein | BD Bioscience | 610787 | 1:1000 | WB |
| alpha synuclein | Leica | ASYN-L | 1:500 | IHC |
| Tau (AT8) | Thermo | MN1020 | 1:500 | IHC |
| Amyloid β (4G8) | Signet | SIG39240 | 1:500 | IHC |
| GFAP | Biomol | GA1170 | 1:10,000 | WB |
| PVALB | Sigma | P3088 | 1:1000 | IHC,WB |
| Calretinin | Sigma | C9848 | 1:1000 | IHC |
| Calbindin | Sigma | C7479 | 1:1000 | IHC |
| GAD65/67 | Santa Cruz | sc-58531 | 1:1000 | IHC, WB |
| VGlut1 | Synaptic Systems | 135311 | 1:1000 | WB |
| Glut-1 | Thermo | PA1-21041 | 1:200 | IHC |
| GluR1/GR1A1 | AbCam | ab31232 | 1:1000 | WB |
