# Supplementary material for: Analysis of primary visual cortex in dementia with Lewy bodies indicates GABAergic involvement associated with recurrent complex visual hallucinations
Source: Acta Neuropathol Commun. 2016 Jun 30;4:66. doi: 10.1186/s40478-016-0334-3 (PMC4928325; doi:10.1186/s40478-016-0334-3)
Supplement: Additional file 3: Table S2. — Case Details For Microarray, q-PCR and Biochemistry Series [2, 3, 41]. (DOC 41 kb) [file 40478_2016_334_MOESM3_ESM.doc]

**Additional file 3: Table S2 Case Details For Microarray, q-PCR and Biochemistry Series**

Age, age at death in years; PMI, post mortem interval (time from death to tissue fixation or freezing); pH, brain pH determined at post mortem on fresh brain tissue; Braak neurofibrillary staging according to (41). Compound Lewy body score is as described using consensus guidelines (2, 3).

|  | Control  N=12 | DLB  N=13 | AD  N=13 |
| --- | --- | --- | --- |
| Age (±SD)/ years | 76.5 (±7.4) | 77.6 (±4.0) | 78.3 (±6.1) |
| PMI (±SD)/ hours | 18.5 (±5.8) | 19.5 (±7.5) | 19.4 (±8.8) |
| Male/Female | 6/6 | 9/4 | 7/6 |
| pH (±SD) | 6.3 (±0.4) | 6.3 (±0.3) | 6.0 (±0.4) |
| Braak Stage(±SD) | 2 (0-4) | 2 (2-4) | 6 (4-6) |
| Lewy Body Score (±SD) | 0 | 7.91 (±8.1) | 0 |
| Eye Disease* (positive/negative) | 1/11 | 6/7 | 2/11 |

†, Braak neurofibrillary tangle staging (41), data are presented as median and (range)

‡, Cortical Lewy body score (3), data are presented as median and (range)

*, significant eye disease: diabetic retinopathy, glaucoma, retinal vein occlusion, macular degeneration, bilateral cataracts, orbital exentoration, papilloedema.
