# Supplementary material for: Analysis of primary visual cortex in dementia with Lewy bodies indicates GABAergic involvement associated with recurrent complex visual hallucinations
Source: Acta Neuropathol Commun. 2016 Jun 30;4:66. doi: 10.1186/s40478-016-0334-3 (PMC4928325; doi:10.1186/s40478-016-0334-3)
Supplement: Additional file 4: Table S5. — Taqman q-RT-PCR Assay Details used in determination of mRNA relative levels in primary visual cortex. Assay ID, specific assay identifier; Gene Name, mRNA target of assay. (DOC 41 kb) [file 40478_2016_334_MOESM4_ESM.doc]

| Assay ID | Gene Symbol | Gene Name | Target Exon(s) | NCBI Gene Reference |
| --- | --- | --- | --- | --- |
| Hs00241471_m1 | GAD1 | glutamate decarboxylase 1 (brain, 67kDa) | 12 | NM_000817.2 |
| Hs00169098_m1 | APP | Amyloid Precursor Protein | 10-11 | NM_000484.3 |
| Hs00326720_s1 | MAPT | microtubule-associated protein tau | 15 | NM_001123066.3 |
| Hs00161045_m1 | PVALB | parvalbumin | 4-5 | NM_002854.2 |
| Hs00268296_m1 | SNAP25 | synaptosomal-associated protein, 25kDa | 1-2 | NM_130811.2 |
| Hs00168219_m1 | GRIN2A | Glutamate Receptor 2 A | 5-6 | NM_000833.3 |
| Hs00240907_m1 | SNCA | Alpha-synuclein | 4-5 | NM_000345.3 |
| Hs00380947_m1 | BDNF | Brain Derived Neuronotrophic Factor | 1-2 | NM_170733.3 |
| Hs00176354_m1 | DLG4 | Post Synaptic Density Protein 95kDa (PSD-95) | 2-3 | NM_001128827.1 |
| Hs00199577_m1 | SYN1 | synapsin I | 5-6 | NM_006950.3 |
| Hs00300531_m1 | SYP | synaptophysin | 3-4 | NM_003179.2 |
| Hs99999905_m1 | GAPDH | glyceraldehyde-3-phosphate dehydrogenase | 3 | NM_002046.4 |
|  |  |  |  |  |

**Additional file 4: Table S5 Taqman q-RT-PCR Assay Details used in determination of mRNA relative levels in primary visual cortex. Assay ID, specific assay identifier; Gene Name, mRNA target of assay.**
