# Supplementary material for: Analysis of primary visual cortex in dementia with Lewy bodies indicates GABAergic involvement associated with recurrent complex visual hallucinations
Source: Acta Neuropathol Commun. 2016 Jun 30;4:66. doi: 10.1186/s40478-016-0334-3 (PMC4928325; doi:10.1186/s40478-016-0334-3)
Supplement: Additional file 5: Table S3. — Analysis of pathology related genes in the primary visual cortex in Dementia with Lewy Bodies and Alzheimer’s disease. (DOC 30 kb) [file 40478_2016_334_MOESM5_ESM.doc]

# Additional file 5: Table S3 Analysis of pathology related genes in the primary visual cortex in Dementia with Lewy Bodies and Alzheimer’s disease.

mRNA levels were determined using validated Taqman assays by real time PCR and proteins determined using western blotting of the appropriate protein band with relative expression levels being normalised to GAPDH mRNA or protein. Values represent the 2^-ΔΔCT^ value for mRNA and protein/GAPDH ratio for the specific protein determined using western blotting. No significant alterations in expression were identified. Values are mean ± SEM.

|  | Control | DLB | AD |
| --- | --- | --- | --- |
| *SNCA* mRNA | 1.00 ± 0.16 | 0.61 ± 0.09 | 1.14 ± 0.41 |
| SNCA protein | 0.68±0.20 | 0.82±0.29 | 0.83±0.29 |
| *MAPT* mRNA | 0.683 ± 0.68 | 0.867 ± 0.87 | 2.150 ± 2.15 |
| *APP* mRNA | 1.579 ± 0.47 | 1.194 ± 0.23 | 1.585 ± 0.29 |
|  |  |  |  |
