# Supplementary figures and images for: Analysis of primary visual cortex in dementia with Lewy bodies indicates GABAergic involvement associated with recurrent complex visual hallucinations
Source: Acta Neuropathol Commun. 2016 Jun 30;4:66. doi: 10.1186/s40478-016-0334-3 (PMC4928325; doi:10.1186/s40478-016-0334-3)

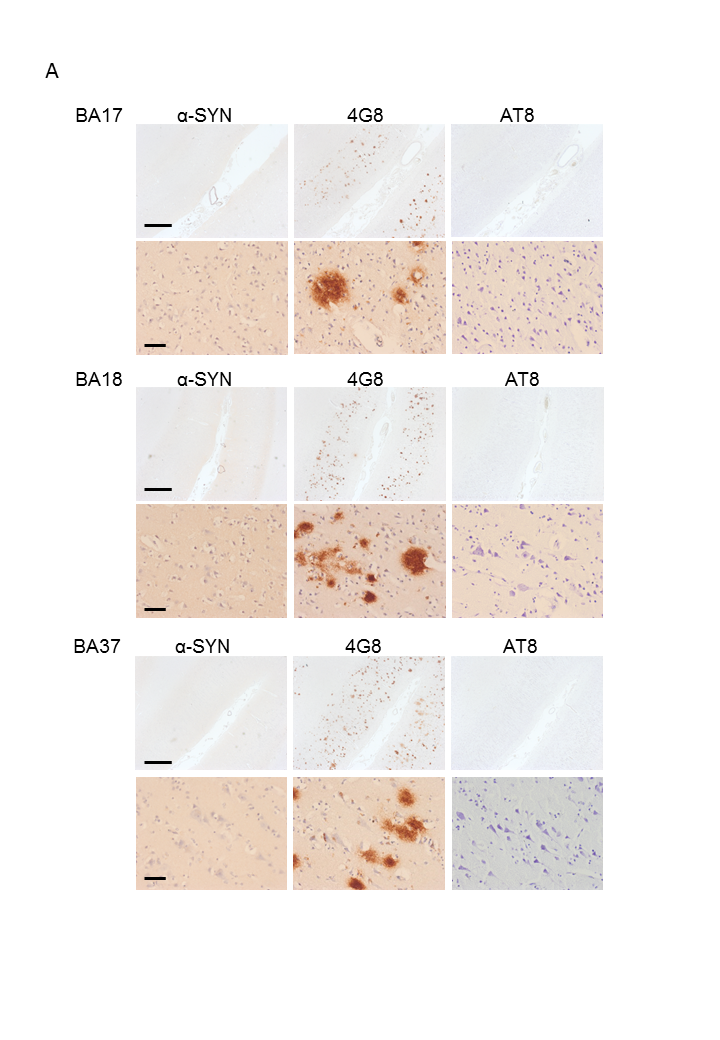

Supplement: Additional file 6: Figure S1. — Neurodegenerative Disease Pathology in the Occipital Lobe in (A) Control and (B) Alzheimer’s disease cases. Representative staining for α-synuclein, Aβ (4G8), and hyperphosphorylated tau (AT8) in primary visual cortex (BA17), secondary visual cortex (BA18), and lateral occipital cortex (BA37) in A) elderly normal control or in B) AD individuals. An absence of α-synuclein pathology was seen in BA17 in either AD or controls cases. Similarly, an absence of AT8 (hyperphosphorylated tau) staining was seen in BA17 in controls, but increasing levels were seen in BA17, BA18 and BA37 in AD. Aβ (4G8 antibody) pathology was present in all cortical regions examined with high levels seen in BA17 in AD cases and in other cortical regions examined. Photomicrographs were taken at x2.5 magnification (upper rows) or at x40 magnification (lower rows) with scale bars at 1000 μm (upper rows) or 50 μm (lower rows). (ZIP 1315 kb) [file 40478_2016_334_MOESM6_ESM.zip › Additional file 7/Supp Figure 1A Khundakar et al Acta Neuropathologica Communications.tif]

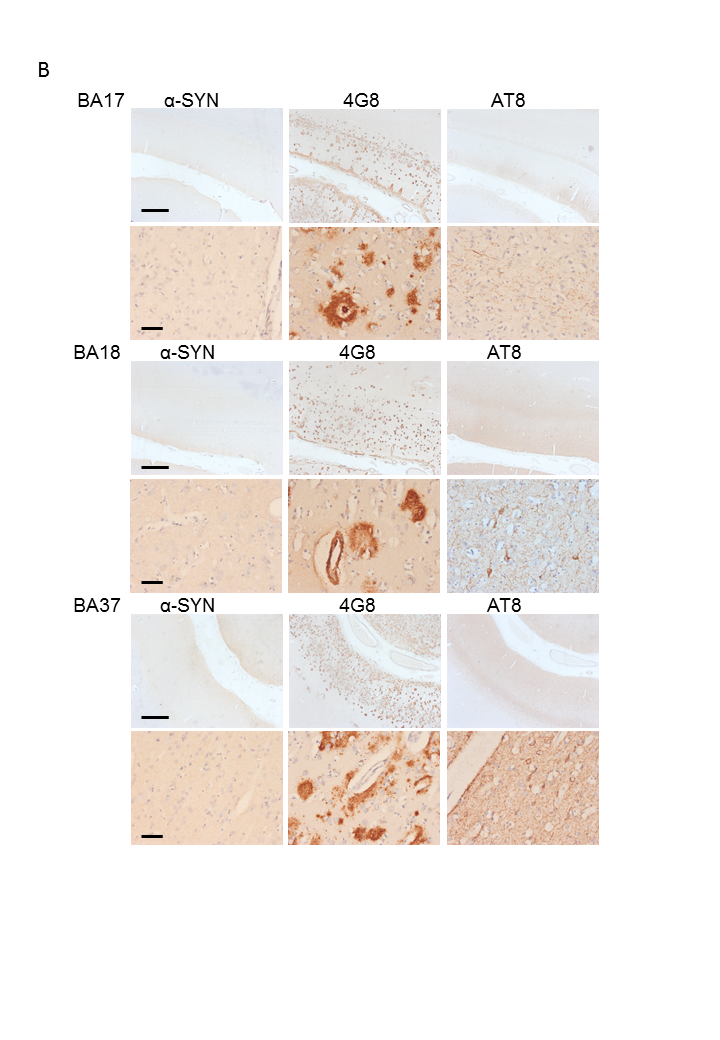

Supplement: Additional file 6: Figure S1. — Neurodegenerative Disease Pathology in the Occipital Lobe in (A) Control and (B) Alzheimer’s disease cases. Representative staining for α-synuclein, Aβ (4G8), and hyperphosphorylated tau (AT8) in primary visual cortex (BA17), secondary visual cortex (BA18), and lateral occipital cortex (BA37) in A) elderly normal control or in B) AD individuals. An absence of α-synuclein pathology was seen in BA17 in either AD or controls cases. Similarly, an absence of AT8 (hyperphosphorylated tau) staining was seen in BA17 in controls, but increasing levels were seen in BA17, BA18 and BA37 in AD. Aβ (4G8 antibody) pathology was present in all cortical regions examined with high levels seen in BA17 in AD cases and in other cortical regions examined. Photomicrographs were taken at x2.5 magnification (upper rows) or at x40 magnification (lower rows) with scale bars at 1000 μm (upper rows) or 50 μm (lower rows). (ZIP 1315 kb) [file 40478_2016_334_MOESM6_ESM.zip › Additional file 7/Supp Figure 1B Khundakar et al Acta Neuropathologica Communications.tif]
