# Supplementary material for: Analysis of primary visual cortex in dementia with Lewy bodies indicates GABAergic involvement associated with recurrent complex visual hallucinations
Source: Acta Neuropathol Commun. 2016 Jun 30;4:66. doi: 10.1186/s40478-016-0334-3 (PMC4928325; doi:10.1186/s40478-016-0334-3)
Supplement: Additional file 7: Table S7. — Functional Systems altered in Primary Visual Cortex in Dementia with Lewy Bodies (DOC 42 kb) [file 40478_2016_334_MOESM7_ESM.doc]

**Additional file 7: Table S7 Functional Systems altered in Primary Visual Cortex in Dementia with Lewy Bodies**

Tables show A) the top 5 Gene Ontology Cellular Component Terms and B) the Kyoto Encyclopaedia of Genes and Genomes (KEGG) biochemical pathways identified through using the set of differentially expressed genes from microarray analysis (see Figure 3 of main text).

A)

| GOCCID | P value | FDR | Count | Size | Term |
| --- | --- | --- | --- | --- | --- |
| GO:0005886 | 3.45e-07 | 7.42e-05 | 32 | 2643 | plasma membrane |
| GO:0071944 | 5.89e-07 | 7.42e-05 | 32 | 2702 | cell periphery |
| GO:0061200 | 7.70e-06 | 4.85e-04 | 3 | 8 | clathrin-sculpted gamma-aminobutyric acid transport vesicle |
| GO:0061202 | 7.70e-06 | 4.85e-04 | 3 | 8 | clathrin-sculpted gamma-aminobutyric acid transport vesicle membrane |
| GO:0060198 | 1.15e-05 | 5.80e-04 | 3 | 9 | clathrin-sculpted vesicle |
|  |  |  |  |  |  |

B)

| KEGG ID | P value | FDR | Count | Size | Term |
| --- | --- | --- | --- | --- | --- |
| 04540 | 1.01e-04 | 8.57e-03 | 5 | 77 | Gap junction |
| 04730 | 2.85e-04 | 9.25e-03 | 4 | 52 | Long-term depression |
| 04720 | 4.35e-04 | 9.25e-03 | 4 | 58 | Long-term potentiation |
| 04970 | 4.35e-04 | 9.25e-03 | 4 | 58 | Salivary secretion |
| 04020 | 8.72e-04 | 1.48e-02 | 5 | 122 | Calcium signaling pathway |
|  |  |  |  |  |  |
