# Supplementary material for: Analysis of primary visual cortex in dementia with Lewy bodies indicates GABAergic involvement associated with recurrent complex visual hallucinations
Source: Acta Neuropathol Commun. 2016 Jun 30;4:66. doi: 10.1186/s40478-016-0334-3 (PMC4928325; doi:10.1186/s40478-016-0334-3)
Supplement: Additional file 8: Table S4. — GAD65/67 Mean (± SD) Cell Counts of GABAergic Neurones in the Primary (BA17) and Secondary (BA18) Visual cortex in Dementia with Lewy Bodies and Alzheimer’s disease. (DOC 30 kb) [file 40478_2016_334_MOESM8_ESM.doc]

**Additional file 8: Table S4 GAD65/67 Mean (± SD) Cell Counts of GABAergic Neurones in the Primary (BA17) and Secondary (BA18) Visual cortex in Dementia with Lewy Bodies and Alzheimer’s disease.**

Whole coronal sections of the occipital lobe were stained with GAD65/67 antisera to identify GABAergic neurones and cell counts through all layers were used to determine total neuronal density. No significant alteration in cell density was seen in either the primary or secondary visual cortex in DLB or AD compared to control.

| Group | Brodmann Area | Density |
| --- | --- | --- |
| Control (N=10) | BA17 | 65.6±10.8 |
|  | BA18 | 46.4±13.8 |
| DLB (N=11) | BA17 | 59.9±18.1 |
|  | BA18 | 37.2±9.3 |
| AD (N=11) | BA17 | 64.9±15.1 |
|  | BA18 | 45.6±10.0 |
|  |  |  |
